# Differential response of lymphatic, venous and arterial endothelial cells to angiopoietin-1 and angiopoietin-2

**DOI:** 10.1186/1471-2121-8-10

**Published:** 2007-03-06

**Authors:** Vicky PKH Nguyen, Stephen H Chen, Jason Trinh, Harold Kim, Brenda L Coomber, Daniel J Dumont

**Affiliations:** 1 Molecular and Cellular Biology Research, Sunnybrook Research Institute, Sunnybrook Health Sciences Centre, Toronto, ON, Canada; 2 Department of Medical Biophysics, University of Toronto, ON, Canada; 3 Ontario Veterinary College, University of Guelph, Guelph, ON, Canada; 4 Heart & Stroke/Richard Lewar Centre of Excellence, Faculty of Medicine, University of Toronto, Toronto, ON, Canada; 5 R. Samuel McLaughlin Centre for Molecular Medicine, Toronto, ON, Canada

## Abstract

**Background:**

The lymphatic system complements the blood circulatory system in absorption and transport of nutrients, and in the maintenance of homeostasis. Angiopoietins 1 and 2 (Ang1 and Ang2) are regulators of both angiogenesis and lymphangiogenesis through the Tek/Tie-2 receptor tyrosine kinase. The response of endothelial cells to stimulation with either Ang1 or Ang2 is thought to be dependent upon the origin of the endothelial cells. In this study, we examined the effects of the angiopoietins on lymphatic, venous and arterial primary endothelial cells (bmLEC, bmVEC and bmAEC, respectively), which were isolated and cultured from bovine mesenteric vessels.

**Results:**

BmLEC, bmVEC and bmAEC cell populations all express Tie-2 and were shown to express the appropriate cellular markers Prox-1, VEGFR3, and Neuropilin-1 that define the particular origin of each preparation. We showed that while bmLECs responded slightly more readily to angiopoietin-2 (Ang2) stimulation, bmVECs and bmAECs were more sensitive to Ang1 stimulation. Furthermore, exposure of bmLECs to Ang2 induced marginally higher levels of proliferation and survival than did exposure to Ang1. However, exposure to Ang1 resulted in higher levels of migration in bmLECs than did to Ang2.

**Conclusion:**

Our results suggest that although both Ang1 and Ang2 can activate the Tie-2 receptor in bmLECs, Ang1 and Ang2 may have distinct roles in mesenteric lymphatic endothelial cells.

## Background

Tek or Tie-2 is the receptor tyrosine kinase (RTK) for the angiopoietin family of ligands (Ang1, 2, 3, and 4). The role of Tie-2 in endothelial cells has been extensively studied over the years, and the discovery of impaired lymphatic vessel patterning and function in Angiopoietin-2 (Ang2) knockout mice has since added extra complexity to this growing field [1]. Tie-2 has also been shown to be expressed in lymphatic endothelial cells [2]. In vivo studies using slow-release pellets of an engineered form of Ang1, cartilage oligomeric matrix protein-Angiopoietin-1 (COMP-Ang1), have further showed induction of angiogenesis and ectopic lymphangiogenesis in mouse cornea [2]. In addition, over-expression of Ang1 in the skin of mouse ears via recombinant adeno-associated virus gene delivery induced lymphatic endothelial cell proliferation, lymphatic vessel enlargement, sprouting, and branching [3].

Prior to the discovery of the involvement of Tie-2 and its ligands in lymphangiogenesis, the role of Tie-2 and it ligands in angiogenesis was the focus of mouse genetic studies. Ang1 deficient mice exhibited phenotypes similar to those of Tie-2 knockout mice. These phenotypes included impaired myocardial trabeculation and endocardial development as well as lack of perivascular recruitment to endothelial cells undergoing angiogenesis [4]. Ang2 was initially characterized as an Ang1 competitive antagonist since transgenic over-expression of Ang2 produced angiogenic defects resembling those of Ang1 or Tie-2 knockout mice [5].

In recent studies, evidence suggesting condition-dependent agonistic roles for Ang2 brought into question the initial characterization of Ang2 as simply a competitive antagonist of Ang1. Ang2 has been shown to activate Tie-2 phosphorylation at high concentrations leading to cell survival via the Akt signalling pathway [6]. Following a 24-hour pre-treatment, Ang2 can lead to vessel growth in a fibrin matrix model [7]. Ang2 can also induce tubule formation in murine brain capillaries and promote endothelial cell migration [8]. In addition to inhibiting JNK/SAPK phosphorylation, Ang2 has been shown to induce phosphorylation of Tie-2, AKT, Erk1/2, and p38 members of the mitogen activated protein kinases in the human umbilical vein endothelial cells (HUVEC) [9]. Other functions of Ang2 in vasculogenesis include promotion of endothelial cell (EC) adhesion independently of Tie-2 [10], regulation of differentiation of cells surrounding the cortical peritubular capillaries of the kidneys [11], and modulation of retinal and hyaloid blood vessel remodelling [12].

Complementing these studies were mouse knockout experiments that further elucidated the impotance of Ang2 in endothelial cell function. Ang2-null mice (strain 129/J) exhibited defects in the hyaloid blood vasculature and gross defects in lymphatic remodelling [1]. Skin oedema in Ang2-null mice correlated with improper recruitment of support cells such as smooth muscle cells responsible for the contractile function of lymphangions in peripheral or dermal lymphatic vessels [1]. The Ang2-null mice also developed lethal chylous ascites and edema in the peritoneal cavity around day 14, correlating with improperly remodelled lacteals in villi, disorganization and hypoplasia of intestinal lymphatic capillaries, and deficient smooth muscle association in mesenteric-collecting lymphatic vessels [1]. The function of Ang2 is therefore not limited to antagonistic regulation of Ang1 in Tie-2 signalling.

In this study, we demonstrate that bovine mesenteric venous, arterial, and lymphatic endothelial cells (bmVEC, bmAEC, and bmLEC) responded differentially to Ang1 and Ang2. Firstly, both Ang1 and Ang2 activated Tie-2 and downstream ERK1/2 in bmLEC. However, whereas Ang1 promoted migration in bmLECs, Ang2 slightly more effectively promoted proliferation and survival in these cells. Secondly, Ang1 more effectively activated Tie-2 and downstream ERK1/2 in bmVECs and bmAECs than did Ang2. However, whereas Ang1 promoted survival, and migration in bmVECs and bmAECs, Ang2 did not stimulate the same responses in these cells. Whereas Ang1 produced a small proliferative response in bmAECs, neither Ang1 nor Ang2 produced a proliferative response in bmVECs. Taken together, our results suggest cellular responses to Ang1 and Ang2 stimulation vary depending on the origin of the endothelial cells.

## Results

### BmLEC isolation and culture

**Figure 1:**
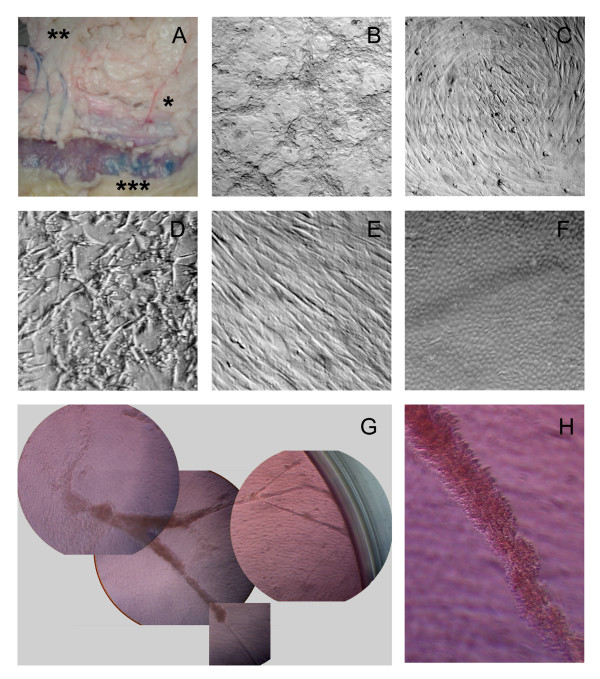
**Isolation of Bovine Mesenteric Lymphatic Endothelial Cells**. A/ Evan's blue dye, when injected into the lymph node of bovine mesentery (***), outlined lymphatic vessels (**), but not blood vessels (*). B/ A mixed cell population did not form a monolayer in culture. C/ A mixed cell population of predominantly smooth muscle cells and fibroblasts formed circular patterns in culture. D/ Typical morphology of a cell population predominantly comprising of fibroblasts. E/ Morphology of a cell population predominantly comprising of smooth muscle cells. F/ Morphology of a cell population predominantly comprising of lymphatic endothelial cells. G/ Mixed cultures of cells extracted from lymphatic vessels spontaneously formed lymphatic tube-like structures with ends attached to the walls of the tissue culture dish. H/ Enlargement of the lymphatic tube-like structure seen in "G".

In order to distinguish translucent lymphatic vessels from surrounding fatty tissue, Evan's blue dye was injected into exposed mesenteric lymph nodes. The blue staining of post-nodal lymphatic vessels facilitated excision and processing of the vessels to extract endothelial cells. Blood vessels remained red after dye injection (Figure 1A) and therefore could not be mistaken for lymphatic vessels. Initial cultures of cells extracted from dispase- and collagenase-treated excised bovine mesentery lymphatic vessels were at least 50% endothelial based on cellular morphology. Cells cultured from treated vessels were a mixture of smooth muscle cells (SMCs), fibroblasts, and cobblestoned bmLECs.

Unlike high-purity endothelial cell populations, a mixed cell population did not form a cobblestone monolayer when grown to confluency (Figure 1B). The morphologies of the different cells within the mixed population were visually distinguishable. A mixed population of predominantly SMCs, fibroblasts, and a few bmLECs formed circular "swirly" patterns (Figure 1C). This morphology has previously been described by Leak and Jones, and Johnston and Walker [13,14]. Populations of cells that predominantly consisted of fibroblasts (Figure 1D) or SMCs (Figure 1E) had distinct morphologies unlike the cobblestoned appearance of bmLEC at confluency (Figure 1F).

To obtain purity greater than 90%, contaminating SMCs and fibroblasts were eliminated from the culture by direct aspiration, differential trypsinization, and by limited dilution. Resulting cultures of the primary cells bmLECs had doubling times of approximately 24 hours [see Additional file 1], consistent with doubling times reported by Leak and Jones [13]. These bmLECs were robust and could be frozen and thawed within 20 passages.

Previously, Leak and Jones [13] described the spontaneous formation of lymphatic tube structures *in vitro *from confluent monolayer of lymphatic endothelial cells. We also observed the formation of these tubes in 96-well plates of bmLEC cell populations left at confluency for at least a few weeks (Figures 1G and 1H). Cells at the ends of the tubes attached to and migrated up the sidewalls of the tissue culture wells (Figure 1G). The formation of lymphatic tubular structures in culture indicated that extracted bmLECs maintain lymphatic phenotypic character.

### Comparing bmLECs to bmVECs, and to bmAECs

**Figure 2:**
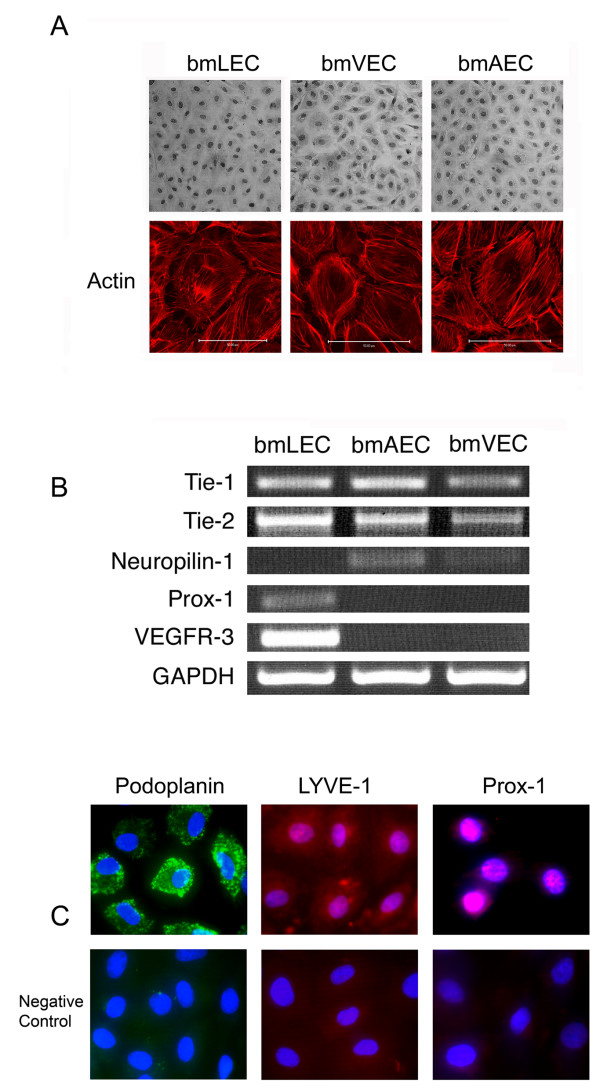
**Similarities and differences between bmLECs, bmVECs and bmAECs**. A/ BmLECs could not be distinguished from bmVECs or bmAECs by cellular morphology alone. Actin staining of the three cells using Rhodamine-phalloidin showed actin filaments arranged in dense bands around the cell periphery and actin stress fibers extending throughout the cytoplasm. B/ Despite being similar in morphology, RT-PCR analysis showed bmLECs and not bmVECs or bmAECs expressed Prox-1 and VEGR-3; bmAECs and not bmVECs expressed neuropilin-1. All cells expressed Tie-2 and Tie-1. C/ By immunofluorescence, bmLECs stained positive for podoplanin, Prox-1, and LYVE-1.

In essence, the strategy used to isolate bmLECs was also used to isolate bmAECs and bmVECs. Dye injection was unnecessary since large mesenteric arteries and veins were discernable from surrounding fatty tissue, unlike milky white lymphatic vessels. Arteries were thick-walled vessels that were easily distinguished from slightly larger and thin-walled veins. Cultured bmLECs, bmVECs, and bmAECs exhibited the same cobblestoned monolayer morphology at confluency (Figure 2A), although the packing of cells together was somewhat different. After the formation of the confluent monolayer, bmAECs, bmVECs, and bmLECs continued to grow at different rates to form tighter-packed monolayer [see Additional file 1]. BmAECs grew fastest at this stage and formed the densest monolayer. BmAECs were followed by bmVECs, which formed the second densest monolayer. BmLECs grew at the slowest rate at this stage and formed the least dense monolayer [see Additional file 1 and data not shown].

Staining the cells with fluorescent phalloidin revealed that the arrangement of actin microfilaments in each cell type appeared similar to each other, typically with dense peripheral bands of actin filaments, and prominent stress fibers throughout the cytoplasm (Figure 2A). However there seemed to be some qualitative differences in the intracellular distribution of these actin filaments. There were extensive dense peripheral bands of actin, with relatively few stress fibers in bmVECs, whereas stress fibers were more prominent in bmAECs. In contrast, bmLECs seemed to have an intermediate combination of both actin filament arrangements (Figure 2A).

Upon achieving confluency, apart from differences in cell packing, bmLECs were indistinguishable from bmVECs and bmAECs by phase contrast microscopy alone (data not shown). However, using gene-specific primers and reverse transcriptase-PCR analysis, and using antibodies specific for Prox-1, LYVE-1, or Podoplanin and immunofluorescence, we were able to demonstrate that these endothelial cell preparations retained their lineage-specific properties in culture. All three cell preparations expressed Tie-1 and Tie-2 (Figure 2B), whereas bmLECs but not blood ECs expressed VEGFR-3 (Flt-4) and Prox-1 (Figures 2B). Immunofluorescence also showed bmLECs to express LYVE-1 and Podoplanin (Figure 2C), both of which have been shown to be specific markers of lymphatic endothelial cells [16]. Furthermore, bmAECs and not bmVECs expressed neuropilin-1, which has been shown to be a specific arterial endothelial cell marker [17] (Figure 2B).

### Activation of Tie-2 in bmECs by Ang2

In order to ensure Tie-2 in bovine cells is sufficiently similar to human Tie-2 to be stimulated with human angiopoietin ligands, amino acid sequences of bovine, mouse, and human Tie-2 were aligned with CLUSTALW [see Additional file 2]. Protein sequence alignment of human, mouse and bovine Tie-2 indicated 95% identity between bovine and human sequences and 92% identity between mouse and human, or mouse and bovine sequences. More importantly, the residues found by Barton et al. [18] to be important for the interaction of Tie-2 with Ang2 were conserved across the three species [see Additional file 2, blue boxes], and all tyrosine residues in the kinase domain were perfectly conserved [see Additional file 2]. Based on this observation, bovine endothelial cells should be highly suitable for stimulation with human ligands.

**Figure 3:**
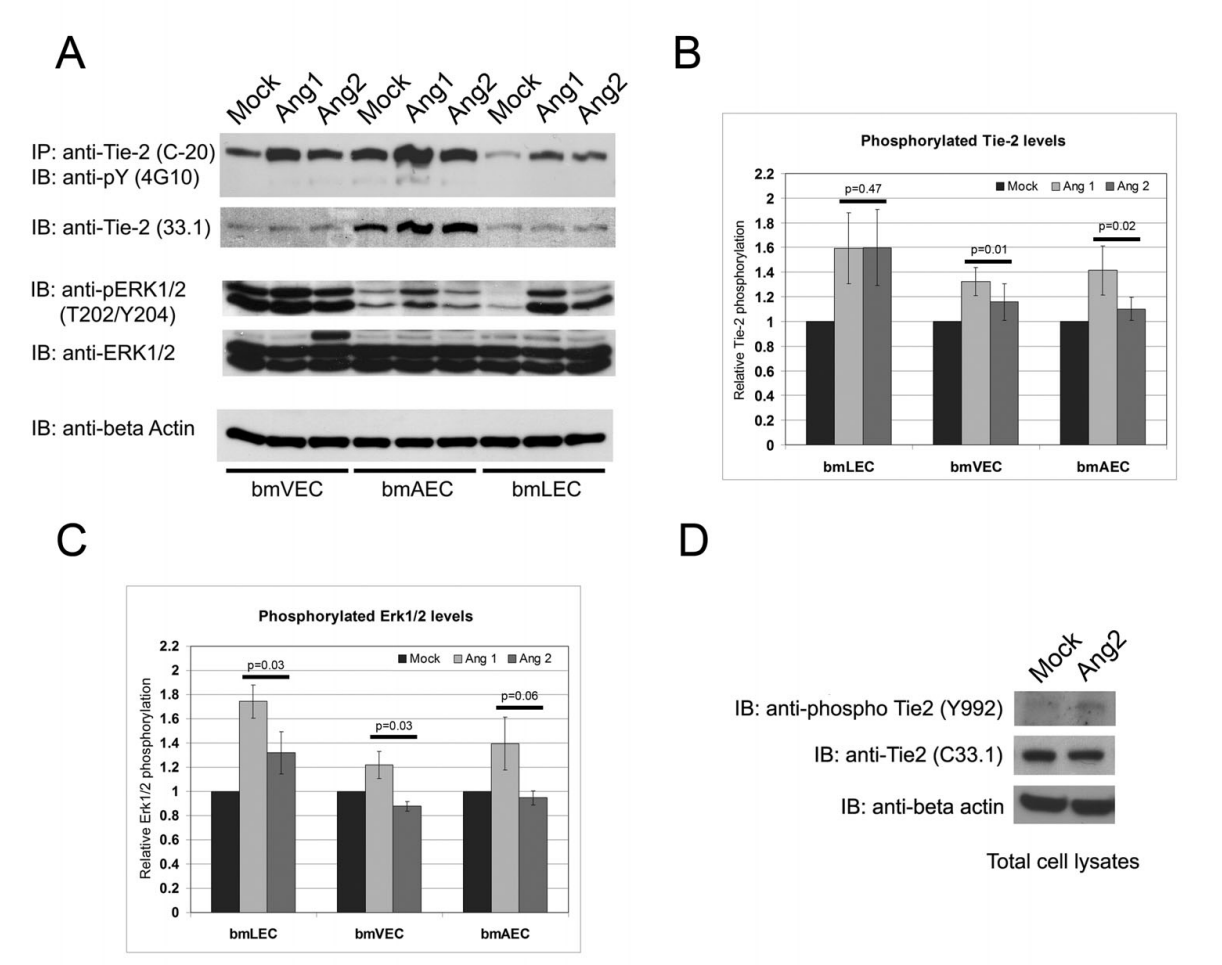
**Stimulation of Tie-2 phosphorylation in bmECs by Ang1 and Ang2**. A/ Stimulation of bmLECs, bmVECs, and bmAECs with 800 ng/mL Ang1 (clustered with anti-His antibody), or 800 ng/mL Ang2, or with clustering antibody alone (Mock). Ang1 stimulation resulted in activation of Tie-2 in bmLECs and to a lesser extent bmAECs and bmVECs. B/ Phosphorylated Tie-2 signals from blots of three independent experiments were measured via densitometry (ImageQuant) and normalized to corresponding total Tie-2 levels. Paired Ang1- and Ang2- stimulated, normalized Tie-2 phosphorylation levels in each experiment were compared by ttest and p-values for 95% CI are shown. Both Ang1 and Ang2 stimulated Tie-2 to comparable levels in bmLECs, whereas Ang1 was more effective in stimulating Tie-2 in bmVECs and bmAECs. C/ Phosphorylated ERK1/2 signals from blots of the same three independent experiments from B/ were measured via densitometry (ImageQuant) and normalized to corresponding total ERK1/2 levels. Paired Ang1- and Ang2-stimulated, normalized ERK1/2 phosphorylation levels in each experiment were compared by ttest and p-values for 95% CI are shown. Ang1 was more effective in stimulating ERK1/2 phosphorylation in all three cell types than Ang2. D/ Stimulation of bmLECs with 800 ng/mL Ang2 resulted in phosphorylation of tyrosine residue 992 on Tie-2.

The Tie-2 receptor protein levels were greatest in bmAECs, whereas the levels in bmVECs and bmLECs were lower (Figure 3A, anti-Tie-2 (33.1)). Once the differences in expression levels of Tie-2 in each cell were accounted for, activation of Tie-2 as judged by its autophosphorylation by Ang2, relative to basal autophosphorylation level (mock treatment), was highest in bmLECs, and was considerably lower in bmAECs and bmVECs (Figure 3A and 3B). Clustered Ang1, which we have previously demonstrated is the most potent form of Ang1 [19], stimulated the activation of Tie-2 in all three cell types above basal levels (Figure 3A and 3B). Of note, the level of phosphorylation of Tie-2 due to Ang1 stimulation as compared to the level due to Ang2 stimulation was the same in bmLECs but different in bmAECs and bmVECs (Figure 3A and 3B).

Studies of HUVEC and capillary endothelial cell systems have shown that Ang1 and Ang2 stimulation resulted in the activation of extracellularly regulated kinase or ERK1/2 [9,20-22]. In our endothelial cell system, whereas endogenous protein levels of ERK1/2 were not different in bmLECs, bmVECs and bmAECs, active phosphorylated ERK 1/2 (phospho-ERK) levels were quite different. Mock-treated bmVECs contained high amounts of phospho-ERK, whose levels increased slightly upon Ang1 stimulation (Figure 3A and 3C). Ang2 stimulation of bmVECs did not activate ERK1/2 according to phosphorylation levels of the protein (Figure 3A and 3C). Although bmAECs had relative low basal levels of phospho-ERK in the mock-treated sample, these cells responded in a similar fashion as bmVECS to Ang1 stimulation. Phospho-ERK levels were increased above basal levels upon Ang1 stimulation, but not upon Ang2 stimulation in bmAECs (Figure 3A and 3C). In contrast to bmAECs and bmVECs, stimulation of bmLECs with either Ang1 or Ang2 resulted in an increase in phospho-ERK levels, although Ang1 seemed to provide a more potent signal (Figure 3A and 3C).

In order to ascertain whether the autophosphorylation of Tie-2 detected in these immunoblots reflected ligand-dependent activation of Tie-2 and not just phosphorylation by another kinase, we immunoblotted membranes from bmLECs stimulated with Ang2 with an activation-specific antibody directed to phosphotyrosine 992. Phosphorylation of tyrosine 992 has been shown to reflect activation of kinase activity in Tie-2 [23]. Tie-2 was phosphorylated at this residue when bmLECs were stimulated with Ang2 (Figure 3B), indicating that kinase activity of the tyrosine receptor was activated.

### BmLEC proliferation is enhanced by Ang1 and Ang2

Blood vasculature endothelial proliferation in response to growth factors is considered a key angiogenic response. In the study of lymphangiogenesis, angiogenesis-related assays would need to be adapted to the study of key lymphangiogenic responses of lymphatic endothelial cells such as proliferation. Thus, we tested the response of bmLECs to different proliferative factors. ^3^H-thymidine uptake assays of bmLECs stimulated with VEGF-A^165^, VEGF-C^C156S^, bFGF, EGF [see Additional file 3] demonstrated that all of the growth factors to varying degrees stimulated thymidine upatke in bmLEC. The greatest response of bmLECs was to bFGF and VEGF-C^C156S ^[see Additional file 3] confirming the phenotypic fidelity of bmLECs *in vitro*.

**Figure 4:**
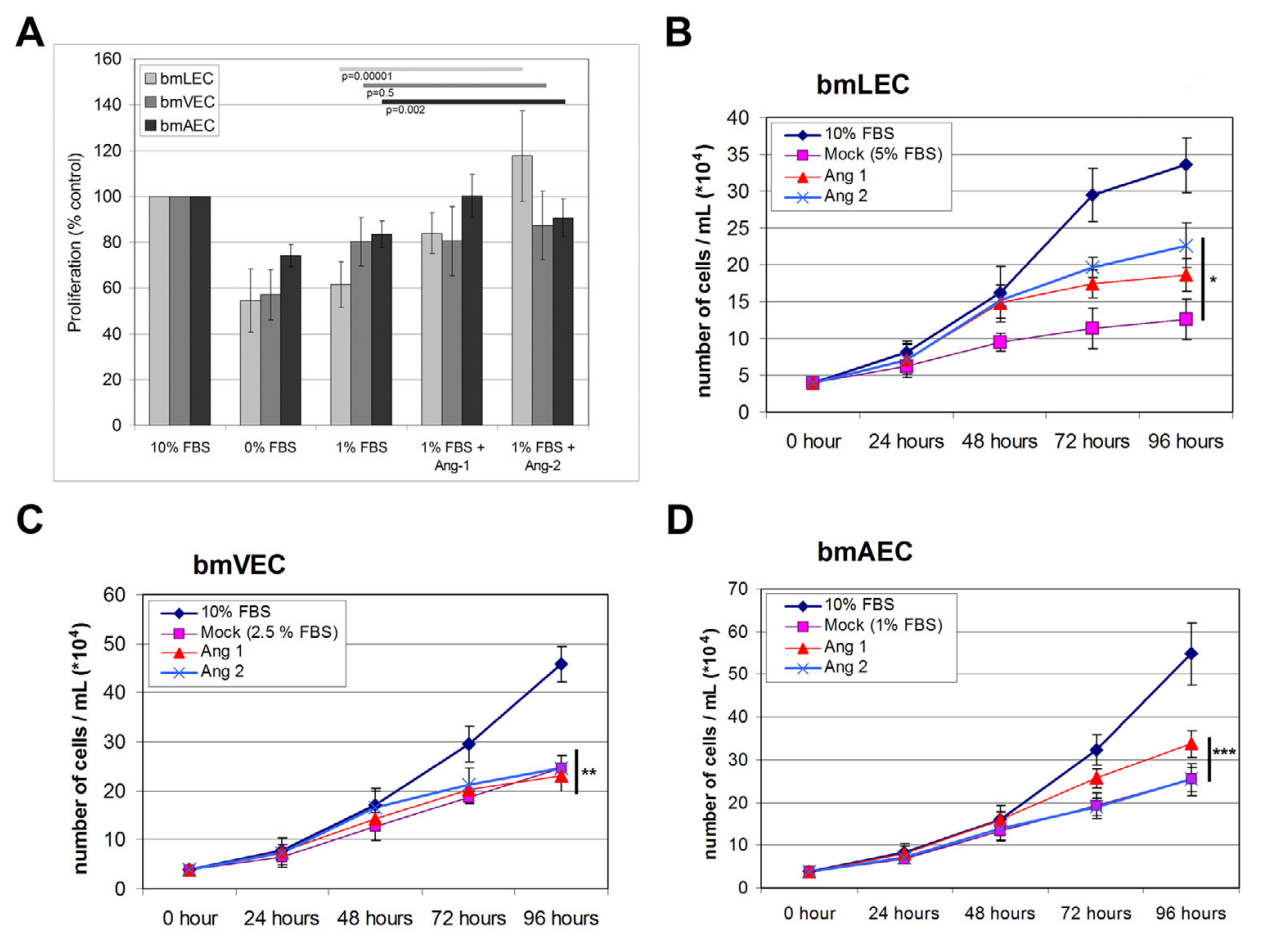
**Proliferation of Ang1 and Ang2 stimulated bmECs**. A/ BmLECs, bmAECs and bmVECs were tested for proliferative response in the presence of Ang1 and Ang2 via tritiated thymidine uptake. Clustered Ang1 (800 ng/mL) and Ang2 (800 ng/mL) did not increase thymidine uptake in bmVECs. Ang1 induced increased thymidine uptake in bmAECs and bmLECs above mock levels (clustering anti-His antibody alone). Ang2-treated bmLECs showed higher levels of thymidine uptake than did Ang1-treated cells. Results from two independent experiments were compiled for the figure. ANOVA p-values for 95% CI are indicated with the colours of the bars corresponding to the colours used to represent cell type. BmLECs (B), bmVEC (C), and bmAEC (D) were tested for proliferative response in the presense of clustered Ang1 (800 ng/mL) and unclustered Ang2 (800 ng/mL) by cell counting. 40 000 cells were seeded (hour 0, approximately 10% confluency) and monitored every 24 hours. Trypan blue exclusion was used to determine cell viability. Results were compiled from one representative experiment of two independent experiments, each done in tripplicate. ANOVA p-values for 95% CI for cell counts at the 96 hour time points are as follows: B/ BmLEC p = 9E-10 (*); C/ BmVEC p = 0.22 (**); D/ BmAEC p = 6E-7 (***).

Studies have shown that Ang1 stimulation results in the activation of ERK1/2 [9,20-22]. Ang1 has also been shown to stimulate proliferation of endothelial cells [21]. To determine whether Ang1 and Ang2 had differential effects on the proliferation of different types of endothelial cells, we tested the levels of thymidine uptake of the three bmECs in the presence of Ang1 and Ang2 (Figures 4A). Although the basal proliferative activity of each cell preparation was somewhat varied: highest in bmAECs, lower in bmVECs, and lowest in bmLECs (data not shown), the differential responses of the cells to these angiogenic factors relative to control levels was detectable. BmVECs did not respond to either Ang1 or Ang2, whereas bmAECs responded best to Ang1 stimulation (Figure 4A). In contrast, bmLECs responded to both Ang1 and Ang2 but best to Ang2-stimulation (Figure 4A). These results suggest that endothelial cell type influences proliferative responses to either Ang1 or Ang2.

In order to make sure Ang1 and Ang2 stimulation did not increase thymidine uptake in bmLECs independently of cell cycle progression and cellular proliferation, we performed cell count experiments (Figure 4B). We also performed cell count experiments for bmVECs and bmAECs with both ligands to ensure reproducibility of the thymidine uptake data (Figure 4C and 4D). Consistent with the trends shown by thymidine uptake assays, Ang1 and Ang2 both provided proliferative signals to bmLECs, increasing bmLEC numbers over the 96-hour period of the assay (Figure 4B). Furthermore, consistent with thymidine uptake results, Ang2 was marginally more effective than Ang1 in increasing cell counts of bmLECs, although the result was not statistically significant (Figure 4B). However, Ang1 and Ang2 increased bmLEC cell counts significantly above mock levels indicating that both Ang1 and Ang2 were mitogenic for bmLECs (Figure 4B).

Consistent with thymidine uptake results, neither Ang1 nor Ang2 provided proliferative signals to bmVECs (Figure 4C). Neither ligand increased cell counts of bmVECs above mock levels over the 96-hour period (Figure 4C). In contrast and consistent with the trends shown by the thymidine uptake results, Ang1 provided a small proliferative signal to bmAECs, whereas Ang2 did not increase bmAEC cell counts above mock levels over the 96-hour period (Figure 4D). Taken together, these cell count results indicate that whether Ang1 and Ang2 are mitogenic depends on endothelial cell type.

### Ang1 promotes bmEC migration

**Figure 5:**
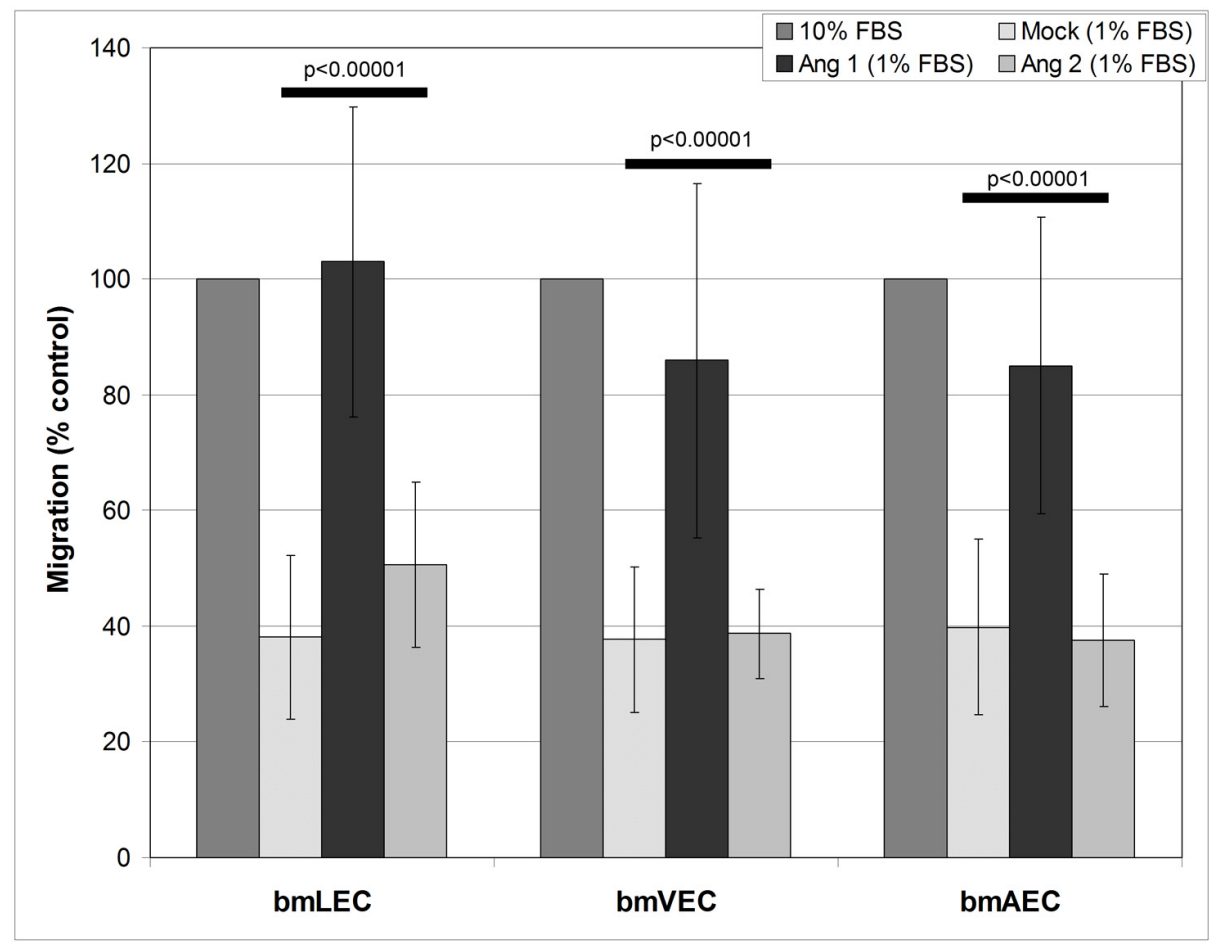
**Migration of Ang1 and Ang2 stimulated bmECs**. BmECs were tested for migration in a modified Boyden chamber assay in the presence of clustered Ang1 (800 ng/mL) and Ang2 (800 ng/mL). Migration of bmLECs, bmVECs, and bmAECs towards clustered Ang1-containing media was significantly increased compared to towards control media (Mock) containing only 1% FBS and clustering anti-His antibody. In contrast, Ang2 did not significantly stimulate migration in any of the cell types, compared to control media. Migration was measured using the Boyden Chamber. Results from two independent experiments were compiled for the figure. ANOVA p-values for 95% CI are indicated.

Endothelial cell migration is another well-accepted criterion for the angiogenic response. Ang1 has previously been shown to induce cell migration, whereas Ang2 has previously been shown not to drive the migration of HUVECs [24]. Using a modified Boyden chamber assay we tested the ability of Ang1 and Ang2 to drive migration of these three cell types. We found that Ang2 did not promote migration of these cells whereas Ang1 was able to drive endothelial cell migration (Figure 5). These results suggest that although bmLECs, bmVECs, and bmAECs responded differentially to Ang1 and Ang2 in proliferative assays, they responded similarly to chemoattractant effects of Ang1.

### Ang2 protects bmLEC from cell death

**Figure 6:**
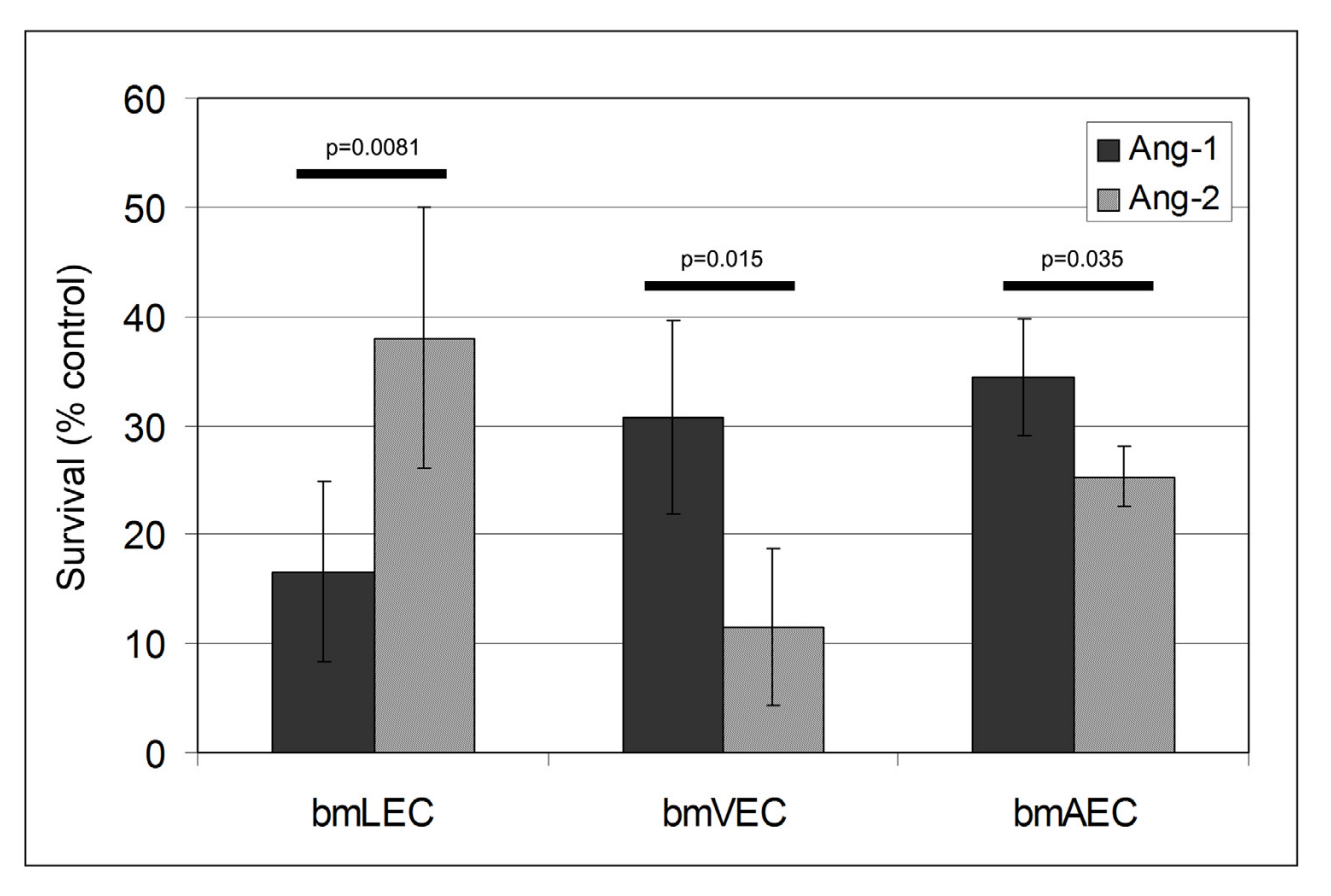
**Survival of Ang1 and Ang2 stimulated bmECs**. Death in bmECs was induced by serum starvation and relative amounts of cell death compared to 0.1%FBS control (0% survival) were measured by quantification of cytoplasmic histone-associated-DNA-fragments (mono- and oligonuclosomes) via ELISA (Roche). Ang2 (800 ng/mL) provided marginally better protection from serum-deprivation-induced cell death than did Ang1 (800 ng/mL) for bmLECs. Ang2 provided marginally worse protection from serum-deprivation-induced cell death than did Ang-1 for bmVECs and bmAECs. Results of three independent experiments were compiled for this figure. P-values of unpaired, two-tailed ttest for 95% CI are indicated.

Many angiogenic factors are known to protect ECs from serum-deprivation-induced cellular apoptosis (reviewed by [25]). Ang1 has previously been shown by numerous groups including ours to protect HUVEC from apoptosis [26-33]. However, a role for Ang2-mediated EC survival remains unclear. Results of some studies suggested that Ang2 counteracts the anti-apoptotic effects of Ang1 and leads to cell death [5,34-36]. Results of other studies suggested that Ang2 mediates a PI3-kinase-dependent cell survival signal [6,9,37]. To investigate the role of Ang2 in serum-deprivation-induced cell death, we tested the ability of Ang1 or Ang2 to promote cell survival on the three cell preparations. Ang1 protected bmVECs and bmAECs from cell death, whereas it had minimal effect on bmLECs (Figure 6). In contrast Ang2 had virtually no effect on bmVECs and a marginal effect on bmAECs (Figure 6). Interestingly, Ang2 produced a dramatic survival response in bmLECs (Figure 6). Together with results from survival and proliferation assays (Summarized in Table 1), these results further suggest that the origin of endothelial cells dramatically influences angiogenesis/lymphangiogenesis-related responses to either Ang1 or Ang2.

**Table 1:** Summary of results

**EC Type**	**Ligand**	**Tie-2/ERK activation**	**Proliferation**	**Survival**	**Migration**
Lymphatic	Ang-1	+/++	+	+	+
	Ang-2	+/+	++	+++	-
Vein	Ang-1	+/+	-	+++	+
	Ang-2	-/-	-	+	-
Arterial	Ang-1	+/+	++	+++	+
	Ang-2	-/-	-	++	-

Table depicts relative levels of Tie-2 and ERK1/2 activation and relative levels of proliferation, survival in serum-deprivation conditions, and migration of Ang1 and Ang2 stimulated bmECs.

## Discussion

In this study, we set out to examine whether the type of EC could affect its response to Ang1 or Ang2. We prepared ECs from lymphatic, venous and arterial vessels taken from bovine mesentery and subjected them to numerous angiogenesis-related *in vitro *assays. Our results demonstrate that the activation of Tie-2 by Ang2 stimulation was as effective as by Ang1 stimulation in bmLECs. In bmVECs and bmAECs, Ang1 stimulation was more effective than Ang2 in activating Tie-2 in bmVECs and bmAECs. The activation of Tie-2 in all three cell types by Ang1 or in bmLECs by Ang2 correlated with the activation of ERK1/2. Ang1 activated ERK1/2 in all three cell types, whereas Ang2 only activated ERK1/2 in bmLECs. Of note, Ang1 seemed to be a more potent activator of ERK1/2 than Ang2 in bmLECs. These results suggest ERK1/2 is a downstream signalling pathway of Tie2 in endothelial cells. Although Ang1 stimulation correlated with ERK1/2 activation in all three cells, Ang1 did not promote proliferation in all three cell types but only weakly in bmAECs and bmLECs (Table 1). Unlike bmVECs, both bmAECs and bmLECs had low levels of basal phosphorylated ERK1/2 levels in the mock samples. This finding suggests that the proliferative response of bmLECs to Ang-2 stimulation may not be solely under the regulation of ERK1/2 downstream of Tie-2. Complex cell-type specific mechanisms present in the endothelial cells suppress the proliferative signal from Ang1 in bmVECs, but promote the weak proliferative responses in bmLEC and bmAECs.

Likewise, additional cell-specific mechanisms may be present in blood endothelial cells that suppress the proliferative and survival signals from Ang2, but are present in lymphatic endothelial cells to promote the proliferative and survival signals from Ang2. This was implied by our findings that Ang2 was not a proliferative signal for bmAECs or bmVECs but only for bmLECs. Similarly, Ang2 did not strongly support serum-free survival of bmVECs and bmAECs but significantly promoted the survival of bmLECs (Table 1). Of note, the level of ERK1/2 activation stimulated by Ang2 in bmLECs was lower than the level stimulated by Ang1, which did not provide as potent a proliferative or survival signal as Ang2 in bmLECs. In summary, our data shows that endothelial cells of different origin have different sets of cellular responses to Ang1 and Ang2 stimulation, suggesting that Ang1 and Ang2 activate distinct sets of signalling molecules in the signalling cascades of endothelial cells of different origin. Thus, we recommend the consideration of Ang2 as a full agonist in endothelial cells of lymphatic origin, but a partial agonist in endothelial cells of blood origin. Since Ang1 and Ang2 also elicited slightly different responses from bmAECs and bmVECs in our study (Table 1), the subtype of endothelial cells of blood origin need to be taken into consideration in future studies of Ang1 and Ang2 signalling and function.

Until recently, Ang2 was thought to be an Ang1 competitive antagonist. This conclusion was made based on the finding that transgenic over-expression of Ang2 produced blood vasculature defects resembling those of Ang1 or Tie-2 knockout mutants [1]. Ang2 competitively blocked activation of Tie-2 by Ang1 but Ang2 was equally as effective as Ang1 in activating Tie-2 phosphorylation in NIH 3T3 fibroblasts ectopically expressing Tie-2 [1]. This finding suggested that endothelial cells have additional components that allow functional discrimination between the two angiopoietin [1]. Our findings clearly indicates that the additional components suggested by these initial studies of Ang2 are endothelial cell-type specific and may be responsible for the agonistic activities of Ang2 in lymphatic endothelial cells. Although there are some functional redundancies, each angiopoietin may be driving a distinct set of cellular events within a specific type of endothelial cell.

Ang2 induction has been shown to associate with progression and prognosis of a variety of human cancers. High expression of Ang2 in malignant tissue correlates with poor survival rate, and/or high frequency of metastasis, and/or high microvascular density (MVD) in patients with advanced colorectal carcinoma, breast cancer, gastric carcinoma, hepatocellular carcinoma (HCC), non-small cell lung cancer (NSCLC), prostate cancer, and ovarian cancer [38-47]. In contrast, expression of Ang1 in these tumours was often found to be at comparatively low to undetectable levels. These studies implicate Ang2 as a potentially potent target of anti-angiogenic cancer therapy. In fact, blocking Ang2 activity by antibodies and specific peptide inhibitors has been efficacious in suppressing tumour growth and reducing endothelial cell proliferation in mice [48].

This evidence suggests that Ang2 is not simply a "context-dependent antagonist/agonist" of Tie-2 signalling; Ang2 can also be a potentially effective candidate target of therapy for pathological conditions involving both angiogenesis and lymphangiogenesis, such as cancer metastasis. Importantly, drug designs intended to target Ang2- while avoiding Ang1-mediated signalling may produce fewer side effects. This point lends justification to further intensive studies to characterize the cell-type specific mechanisms responsible for differential responsiveness to Ang1 and Ang2 stimulation in angiogenesis as well as in lymphangiogenesis. Cells systems such as the one described in this study would be useful tools in this endeavour.

Historically, the angiogenesis field has advanced faster and farther than that of lymphangiogenesis. The discovery of lymphatic markers and the emerging evidence implicating the lymphatic system's central role in a variety of pathological conditions has attracted research interest and driven the field forward. One important question emerges as researchers are confronted with the design of pharmaceuticals in enhancing or mitigating lymphangiogenesis: whether it is possible to specifically target one vascular system without adversely compromising the functions of other vascular system when there is evidence of shared molecular regulators such as the angiopoietins behaving differently in the two systems. Addressing this important question requires *in vitro *systems such as the one described here, that allows simultaneous characterization of the signalling pathways downstream of the angiopoietins in each of the three cell types: venous, arterial and lymphatic endothelial cells.

## Conclusion

We have isolated primary endothelial cells from lymphatic (bmLECs), vein (bmVECs), and arterial (bmAECs) vessels of bovine mesentery. The cells express specific markers of their respective lineages, and were used to test the hypothesis that endothelial cell type determined cellular response such as proliferation, migration, and survival to Ang1 and Ang2 stimulation. We found that indeed, while Ang1 was a chemoattractant for all three cells, Ang1 was a more potent survival signal than Ang2 in bmAEC and bmVEC. In contrast, Ang2 was a more potent survival and proliferative signal than Ang1 in bmLECs. Our data suggests that each endothelial cell type possesses a unique repertoire of signalling proteins that allows that cell type to differentially respond to the angiopoietins.

## Methods

### bmLEC isolation and culture

Methods for isolation and culture of bovine endothelial cells were adapted from previously established methods [13,14]. Sections of the gut mesentery were taken from freshly slaughtered cattle at a local abattoir (Better Beef, Guelph, Ontario). The mesenteric sections were brought back to the laboratory in warm PBS. Connective and fatty tissues, which were superficially rinsed with 75% ethanol, were removed to expose lymph nodes and vessels. Sterile 0.1% Evan's Blue dye was injected into the lymph nodes to high light the lymphatic vessels. Dye was flushed from excised vessels with warm PBS supplemented with penicillin (100 Units/mL, Life Technologies), streptomycin (100 μg/mL, Life Technologies), and fungizone (2.5 μg/mL Gibco, Invitrogen). Distal ends of excised vessels were occluded with surgical suture in order to infuse a solution of dispase I (2.6 U/mL, Roche) and collagenase A (1 mg/mL, Roche). Vessels occluded at both ends to trap the enzyme solution were incubated in PBS at 37°C for 10 minutes. Cells dislodged by the treatment were grown on plates coated with 1% bovine gelatin (Sigma-Aldrich), in DMEM (Dulbecco's modified eagle's medium, Sigma-Aldrich), and standard conditions. Cells isolated from four animals and several vessels from each animal were used in these studies.

### bmVEC and bmAEC isolation and culture

Isolation of cells from venous and arterial vessels from bovine mesentery followed a similar strategy as described for bmLECs. However, the use of dye was unnecessary. Cells isolated from four animals and several vessels from each animal were used in these studies.

### Eliminating contaminating cells

In order to establish high-purity cultures of bovine mesenteric endothelial cells (bmECs), a combination of strategies were used. Firstly, cells that were uninhibited by contact and grew atop the monolayer of endothelial cells were removed by several washes with trypsin such that the monolayer underneath was not disturbed. Secondly, visible regions of the monolayer that did not appear cobblestoned at confluency were removed by direct aspiration. Finally, limited dilution into 96-well plates was used to isolate groups of homogenous endothelial cells. Groups of cells comprising of more than 90% endothelial cells were used in experiments. 90% estimates of purity were based on monolayer cobblestone appearance.

### Detection of transcripts by RT-PCR

Total RNA was prepared from cells isolated from bovine gut mesentery using Tri-Reagent (Sigma-Aldrich) according to manufacturer's instructions. cDNA was synthesized from 1 μg of total RNA with Thermoscript reverse transcriptase (Invitrogen) according to manufacturer's instructions. PCR was performed with Taq polymerase (Qiagen) with primer sequences: *Tie-1 *(forward primer 5'-TGA CTT TGC GGG AGA ACT GG-3', reverse primer 5'-CTC CGA CCA GCA CGT TTC GG-3'), *Tie-2 *(forward primer 5'-GAT TTT GGA TTG TCC CGA GGT CAA G-3', reverse primer 5'-CAC CAA TAT CTG GGC AAA TGA TGG-3'), *Neuropilin-1 *(forward primer 5'-CAG AAC GCT GCC CAC TGC AT-3', reverse primer 5'-CTT TCT GGG TCC TTT TTA TC3'), *VEGFR3 *(forward primer 5'-CGG TGC CCA GTG CGT GGG ACG-3', reverse primer 5'-TTG ACT AGC CAT CGT AGG ACA-3'), *Prox-1 *(forward primer 5'-TTG TCA CCC AAT CAC TTG AAA-3', reverse primer 5'-CTT CCA GGA AGG ATC AAC ATC-3') and *GAPDH *(forward 5'-ACC ACA GTC CAT GCC ATC AC-3', reverse primer 5'-TCC ACC ACC CTG TTG CTG TA-3')

### Cell imaging

bmECs were grown to confluency on gelatin-coated glass coverslips, fixed with 4% paraformaldehyde in PBS for 10 minutes at room temperature, and permeabilized with 0.1% TritonX-100 (Sigma-Aldrich) for 1 minute at room temperature. Fixed and permeabilized cells were stained with haematoxylin and eosin (H&E) or with immunofluorescence using antibodies: Prox-1(RDI), LYVE-1 (RDI), and podoplanin. The following protocol was used for Rhodamine-Phalloidin staining: Fixed and permeabilized cells were incubated in Rhodamine-phalloidin (Molecular Probes, 1:40 dilution) in PBS for 15 minutes, and washed. Cells were mounted on microscope slides with aqua-polymount (Polysciences Inc.) or DAPI-containing Vectashield mounting medium (Vector Laboratories) where appropriate. Bright-field microscope images were produced with the Zeiss Axioplan 2 light microscope (Carl Zeiss). Confocal images were produced using Carl Zeiss LSM 510 laser scanning confocal microscope. Fluorescent images were produced using the Zeiss Axiovert 200 M.

### Stimulation of bovine endothelial cells

BmECs grown on gelatinized plates were moved to bare plates 24 hours prior to stimulation. Cells were stimulated for 15 minutes with human recombinant Ang1 or Ang2 (800 ng/mL, R&D Systems) in 10% FBS. For stimulations with recombinant Ang1, which contains a polyhistidine tag, anti-polyhistidine monoclonal antibody (anti-His, R&D Systems) was used to cluster the ligand according to manufacturer's instructions (ligand to antibody ratio 1:20). Where indicated, mock treatment refers to cells incubated with anti-His antibody alone. Three independent experiments were used to compile the quantitative results of figure 3.

### Cell Lysis and Tie-2 Immunoprecipitation

Bovine ECs were washed twice with ice-cold PBS supplemented with 2 mM activated sodium orthovanadate (Sigma-Aldrich, see [15] for activation protocol). Cells were lysed on ice for 30 minutes with RIPA lysis buffer supplemented with 2 mM sodium orthovanadate, and complete protease inhibitors (Roche). Tie-2 was immunoprecipitated from equal protein amounts of cleared whole cell lysates with 2 μg of anti-Tie-2 antibody C-20 (Santa Cruz Biotechnology) pre-coupled to 25 μl protein A-sepharose (Amersham Biosciences) for 1 hour at 4°C.

### Immunoblotting

Proteins were resolved on 10% PAGE gels and transferred to PVDF (Perkin Elmer) membranes. Antibodies used in immunoblots were: anti- phosphotyrosine 4G10 antibody (1 μg/ml, Upstate Biotechnologies), phosphoTie-2-specific anti-pTyr992 antibody (1:1000, Cell Signalling), anti-Tie-2 antibody 33.1 (0.5 μg/ml, BD Biosciences Pharmingen), and phospho and pan Erk1/2 (44/42 MAPK, 1:1000, Cell Signalling).

### ^3^H-Thymidine uptake assay

Bovine ECs were seeded in 96-well plates at a density of 4500 cells/well in 100 μL 10% FBS DMEM. After 24 hours, 10% FBS DMEM was replaced with 1% FBS DMEM + anti-His antibody (16 ug/mL, "Mock"), with 1% FBS DMEM + Ang1 (800 ng/mL) clustered with anti-His antibody (1:20 ligand to antibody ratio), or with 1% FBS DMEM + Ang2 (800 ng/mL). Cells treated thus were pulsed for 6 hours with 2 μCi of 3H-thymidine (Amersham). After 6 hours, the 96-well plates were placed in -80°C for cell lysis before ^3^H-thymidine incorporation was measured with the TopCount NXT Microplate Scintillation and Luminescence Counter (Packard). ^3^H-thymidine incorporation by bmLECs stimulated by various growth factors: bFGF (1 ng/mL, Sigma-Alrich), EGF (5 μg/mL, Invitrogen), VEGF-A^165 ^(10 ng/mL, R&D Systems), and VEGF-C^Cys156Ser ^(1 μg/mL, R&D Systems) were determined using the same procedure. Results from two independent experiments, each with four replicates were compiled for figure 4A.

### Cell count assay

40 000 cells were plated in each well of 6-well plates at the start of the assay in 10%FBS media or in mock media supplemented with 800 ng/mL supper-clustered Ang1 or 800 ng/mL Ang2 and cell numbers were monitored every 24 hours for 96 hours via cell counting with the haemocytometer. Mock media of bmLECs consisted of 5% FBS, of bmVECs consisted of 2.5% FBS, and of bmAECs consisted of 1% FBS. These percentages of FBS were required to maintain the respective cells over 96 hours while maintaining a detectable level of cellular proliferation and minimizing cell death. All mock media contained 16 ug/mL clustering anti-His antibody. Results of figure 4B–D were compiled from one representative experiment of two independent experiments, each done in tripplicate. Both experiments showed the same results (data not shown).

### Modified Boyden Chamber Migration Assay

Bovine ECs were grown for 24 hours before they were serum starved for 2 hours in 0.1% FBS DMEM. Serum starved bovine ECs were then trypsinized and seeded in 500 μL of 0.1% FBS DMEM at a density of 84 000 cells/well in the upper chamber of 6 well plates containing 8 μm pore-size inserts (Falcon). 1.5 mL of the following were placed in the bottom wells: 10% FBS DMEM (positive control); 0.1% FBS DMEM + anti-His antibody (16 μg/mL, "Mock"); 0.1% FBS DMEM + Ang1 (800 ng/mL) clustered with anti-His antibody (1:20 ligand to antibody ratio); 0.1% FBS DMEM + Ang2 (800 ng/mL,). BmECs migration occurred over a 4-hour time period. Membrane of inserts were fixed and stained with filtered Harris' haematoxilin prior to mounting on microscope slides with aqua-polymount (Polysciences Inc.). Results from two independent experiments were compiled for figure 5, ten random fields were counted for each of three replicates of each experiment.

### Cell death ELISA

Bovine ECs were seeded in 6-well plates at a density of 100 000 cells/plate and grown for 24 hours in standard tissue culture conditions and DMEM supplemented with 10% FBS before the start of the assay. To examine cell death due to serum starvation, 10% FBS DMEM was replaced with 0.1% FBS DMEM + anti-His (16 μg/mL), with 0.1% FBS DMEM + Ang1 (800 ng/mL, clustered with anti-His antibody), or with 0.1% FBS DMEM + Ang2 (800 ng/mL). In order to achieve detectable levels of cell death, bmLECs were harvested after 24 hour serum starvation, bmVECs after 48 hours, and bmAECs after 72 hours. Cell death was determined by quantifying cytoplasmic histone-associated-DNA-fragments (mono- and oligonuclosomes) via the ELISA (enzyme-linked immunosorbent assay, Cell Death ELISA^plus^, Roche) according to manufacturer's specifications. Results of three independent experiments each done in tripplicate were compiled for figure 6.

### Statistic Analysis

Paired, one-tailed t-tests were performed for the x-ray film densitometry results of the three independent experiments compiled for figures 3B and 3C. These parameters were appropriate since compared samples were not randomly selected as in proliferation, survival and migration assays. Compared samples belonged to the same x-ray film with the same film exposure time and linearity, which were different in each of the three experiments. Furthermore, trends of Ang1 being more effective in activating Tie-2 in bmVECs and bmAECs, and in activating ERK1/2 in the three cells remained constant in all three experiments (data not shown). Unpaired, two-tailed t-tests were performed on 95% confidence intervals for the survival assay presented in figure 6. ANOVA was performed when ttest analysis of more than two samples was required as in the proliferation and migration assays presented in figures 4 and 5.

## Authors' contributions

VN (together with DD) conceived of and designed the study, VN drafted the manuscript, carried out the isolation and characterization of bmECs, and carried out all experiments involving the stimulation of bmECs including immunoblots, proliferation, migration, survival assays, and performed all statistical analysis. SC participated in characterization of bmECs, in migration assays, and in the drafting of the manuscript. JT carried out reverse transcriptase PCR experiments and participated in the drafting of the manuscript. HK designed primers for PCR experiments and helped to conceive of the study. BC participated in the isolation of bmECs, and in the drafting of the manuscipt. DD conceived of, designed, and coordinated the study, and participated in the drafting of the manuscript. All authors read and approved the final manuscript.

## Supplementary Material

Additional File 1 **Cell counts of bmECs**. Endothelial cells of each type (bmLEC, bmVEC, and bmAEC) were seeded in equal numbers (approximately 30–40% confluency) and counted every 24 hours. Trypan blue exclusion was used to determine cell viability. Numbers from three independent counts were used to compile the figure. Click here for file

Additional File 2 **Sequence alignment of bovine, murine, and human Tie-2 sequences**. Amino acid sequences were obtained from Uniprot/Swiss-prot and aligned with CLUSTALW [49]: Human Q02763, Bovine Q06807, Mouse Q02858. Boxed in blue are residues found to be important for Ang2 interaction with Tie-2 [18], all of which are conserved between the three species. The transmembrane domain is indicated by the black bar. All tyrosine residues in the cytoplasmic domain of Tie-2 are well conserved between the three species. Click here for file

Additional File 3 **Proliferative response of bmLECs to various growth factors**. Compared to mock-treated bmLECs (anti-His clustering antibody), bmLECs treated with VEGF-A, VEGF-C^Cys156Ser^, bFGF, and EGF, showed statistically significant increases in proliferation as displayed by ^3^H-thymidine uptake in 1% FBS. Results of two independent experiments were compiled for the figure. P-values from unpaired, two-tailed ttests for 95% CI are shown. Click here for file
