# Supplementary figures and images for: Differential response of lymphatic, venous and arterial endothelial cells to angiopoietin-1 and angiopoietin-2
Source: BMC Cell Biol. 2007 Mar 6;8:10. doi: 10.1186/1471-2121-8-10 (PMC1828055; doi:10.1186/1471-2121-8-10)

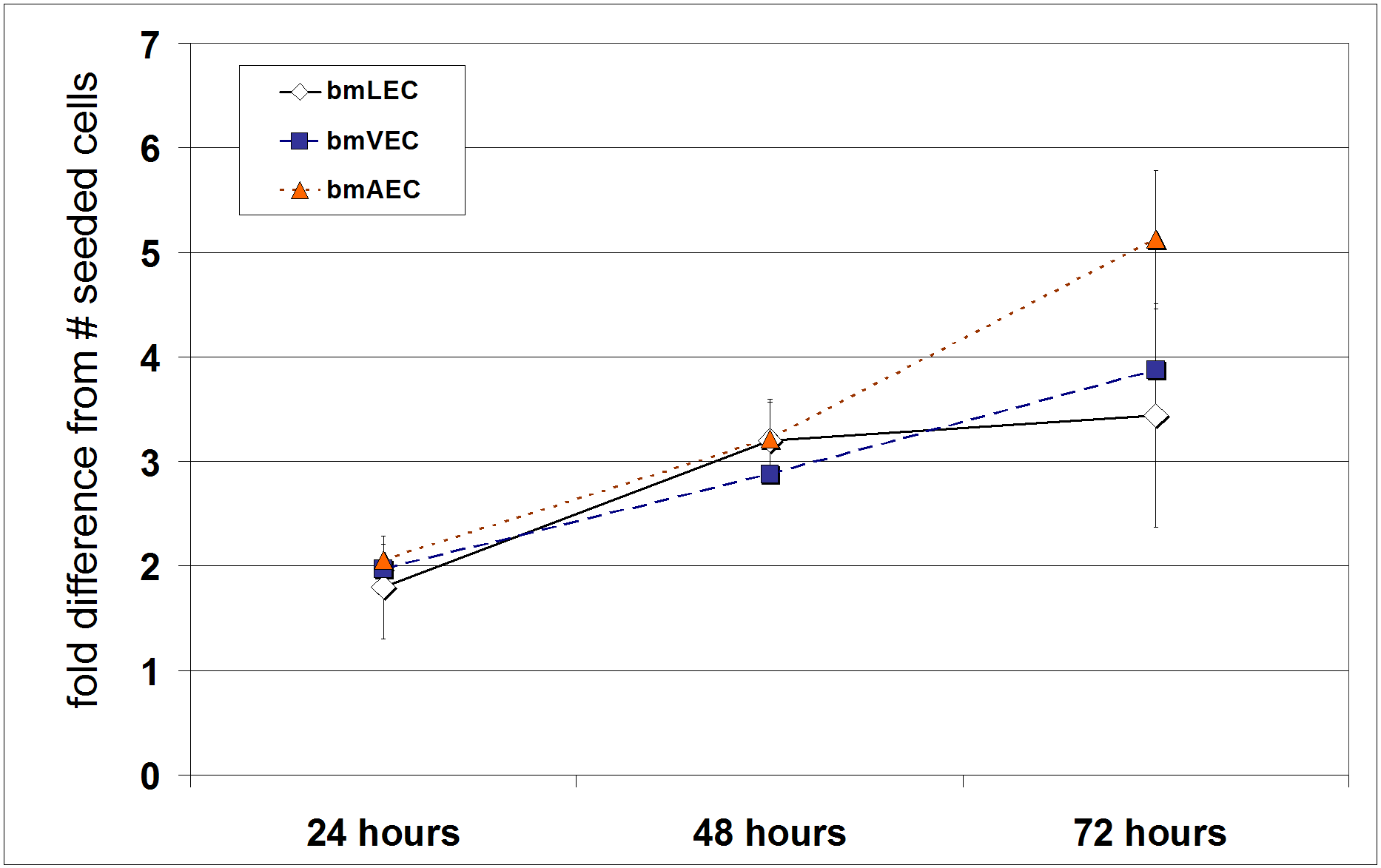

Supplement: Additional File 1 — Cell counts of bmECs. Endothelial cells of each type (bmLEC, bmVEC, and bmAEC) were seeded in equal numbers (approximately 30–40% confluency) and counted every 24 hours. Trypan blue exclusion was used to determine cell viability. Numbers from three independent counts were used to compile the figure. [file 1471-2121-8-10-S1.tiff]

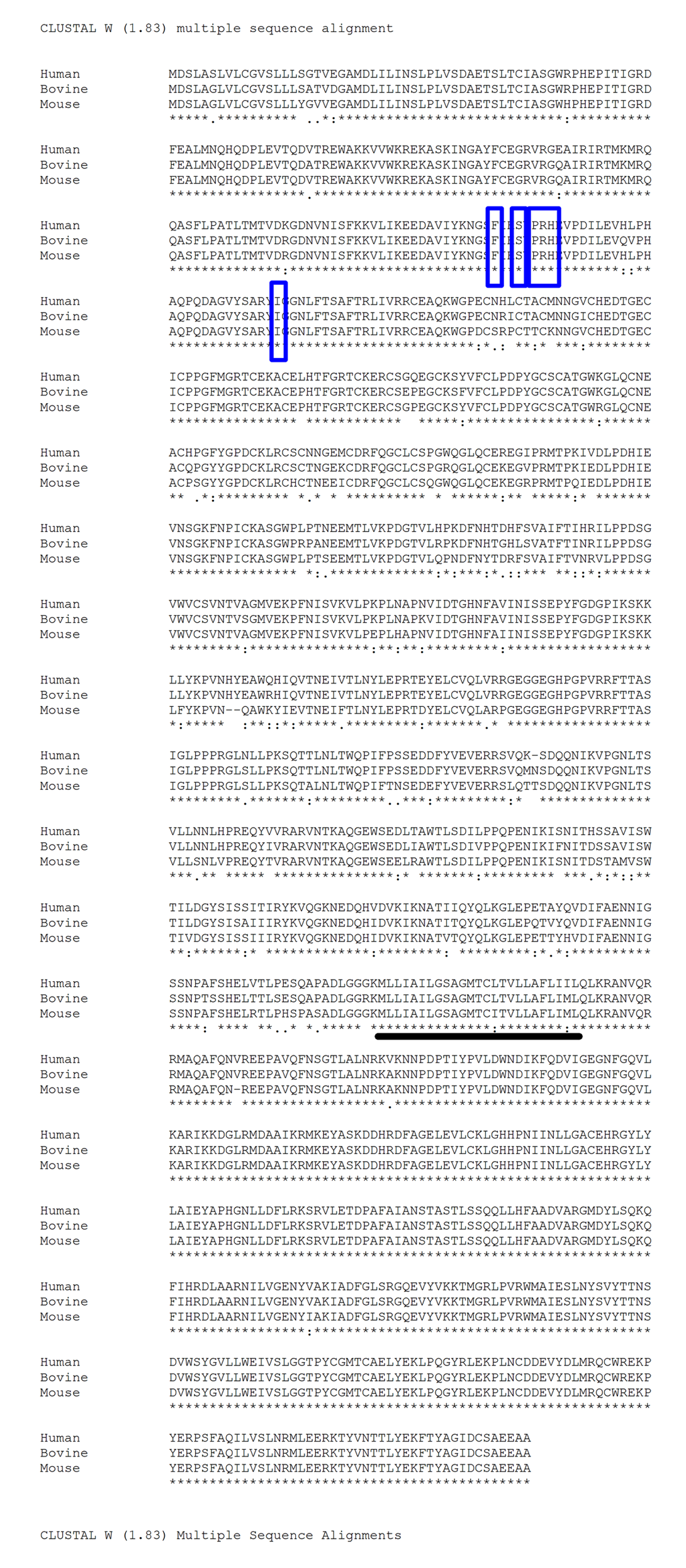

Supplement: Additional File 2 — Sequence alignment of bovine, murine, and human Tie-2 sequences. Amino acid sequences were obtained from Uniprot/Swiss-prot and aligned with CLUSTALW [49]: Human Q02763, Bovine Q06807, Mouse Q02858. Boxed in blue are residues found to be important for Ang2 interaction with Tie-2 [18], all of which are conserved between the three species. The transmembrane domain is indicated by the black bar. All tyrosine residues in the cytoplasmic domain of Tie-2 are well conserved between the three species. [file 1471-2121-8-10-S2.tiff]

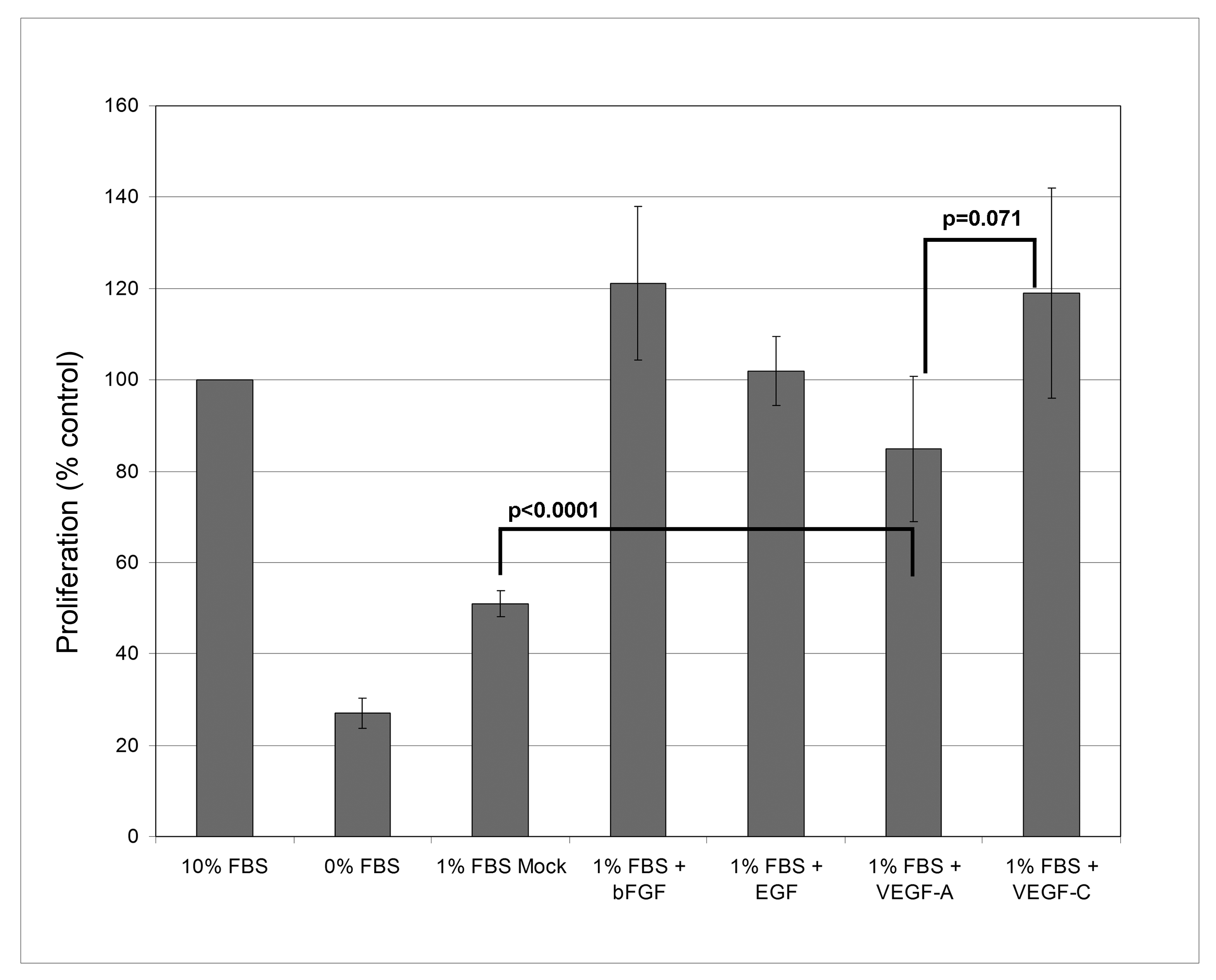

Supplement: Additional File 3 — Proliferative response of bmLECs to various growth factors. Compared to mock-treated bmLECs (anti-His clustering antibody), bmLECs treated with VEGF-A, VEGF-CCys156Ser, bFGF, and EGF, showed statistically significant increases in proliferation as displayed by 3H-thymidine uptake in 1% FBS. Results of two independent experiments were compiled for the figure. P-values from unpaired, two-tailed ttests for 95% CI are shown. [file 1471-2121-8-10-S3.tiff]
